# Curcumin Inhibits Imiquimod-Induced Psoriasis-Like Inflammation by Inhibiting IL-1beta and IL-6 Production in Mice

**DOI:** 10.1371/journal.pone.0067078

**Published:** 2013-06-25

**Authors:** Jun Sun, Yi Zhao, Jinhong Hu

**Affiliations:** Department of Pharmacy, Changhai Hospital, the Second Military Medical University, Shanghai, China; Albert Einstein College of Medicine, United States of America

## Abstract

Curcumin, a selective phosphorylase kinase inhibitor, is a naturally occurring phytochemical present in turmeric. Curcumin has been confirmed to have anti-inflammatory properties in addition to the ability to decrease the expression of pro-inflammatory cytokines in keratinocytes. The interleukin-23 (IL-23)/IL-17A cytokine axis plays a critical role in the pathogenesis of psoriasis. Here, we report that topical use of a curcumin gel formulation strongly inhibited imiquimod (IMQ)-induced psoriasis-like inflammation, the development of which was based on the IL-23/IL-17A axis. IMQ-induced epidermal hyperplasia and inflammation in BALB/c mouse ear was significantly inhibited following curcumin treatment. Real-time PCR showed that mRNA levels of IL-17A, IL-17F, IL-22, IL-1β, IL-6 and TNF-α cytokines were decreased significantly by curcumin in ear skin, an effect similar to that of clobetasol. In addition, we found that curcumin may enhance the proliferation of epidermis γδ T cells but inhibit dermal γδ T cell proliferation. We inferred that curcumin was capable of impacting the IL-23/IL-17A axis by inhibiting IL-1β/IL-6 and then indirectly down-regulating IL-17A/IL-22 production. In conclusion, curcumin can relieve the IMQ-induced psoriasis-like inflammation in a mouse model, similar to the effects of clobetasol. Therefore, we have every reason to expect that curcumin will be used in the treatment of psoriasis in the future.

## Introduction

Psoriasis is one of the most common immune-mediated chronic inflammatory skin disorders characterized by hyperproliferative keratinocytes and massive infiltration of leukocytes [1]. It affects approximately 25 million people in North America and Europe and is likely the most prevalent immune-mediated skin disease in adults. Although the pathogenesis of psoriasis is not fully understood, there is growing evidence to indicate that the interleukin-23 (IL-23)/IL-17A cytokine axis plays a critical role in the disease development [2]–[5]. Evidence suggests that dermal γδ T cells are the major source of IL-17A upon IL-23 stimulation in the skin [2]. Many immune-derived cytokines, including IL-23, IL-17A, IL-20, IL-22, IL-1β, IL-6, and TNF-a, are involved and interact as a network in the pathogenesis of psoriasis.

As a phosphorylase kinase inhibitor, curcumin exhibits anti-oxidant, anti-inflammatory, anti-microbial and anti-carcinogenic activities [6]. Molecular and cellular pharmacological research on curcumin has shown that it has inhibitory effects on NF-κB, MAPK, and cytokines [7]–[9]. For example, it down-regulated the expression of various proinflammatory cytokines (TNF-a, IL-1, IL-2, IL-6, IL-8, and IL-12) by inactivating NF-κB [10], [11]. Curcumin also suppresses the phosphorylation and nuclear translocation of signal transducers and activators of transcription (STATs), including STAT1, STAT3, and STAT4 [12]–14. It is thus possible that curcumin inhibits cytokines, NF-κB and the activation STAT3, which contribute to inflammation and keratinocyte proliferation in psoriasis. All of the above suggests that curcumin is of potential value for the treatment of psoriasis.

Experimental data show that imiquimod (IMQ)-induced dermatitis in mice closely resembles human psoriasis lesions not only with regard to phenotypic and histological characteristics but also in the development of the lesions, which is based on the IL-23/IL-17A axis [15]. This study was designed to investigate the effect of a curcumin gel on an IMQ-induced psoriasis-like mouse model. In doing so, we expected to obtain supportive evidence of a role for curcumin in treating psoriasis.

## Materials and Methods

### Ethics Statement

The protocol (Protocol Number: AE2012-0189) were approved by the experimental animal ethics committee of the Second Military Medical University and met the national guidelines for the care and use of experimental animals. The experimental animal center of the Second Military Medical University was certified by the “International Association for Assessment and Accreditation of Laboratory Animal Care International”.

### Chemicals

The chemicals used in this study include curcumin (Sigma, St. Louis, MO, USA), azone (Shanghai Health-well Chemical, China), hydroxypropylcellulose (HPC)-MF (Hercules Incorporated-Aqualon Division, USA), 5% imiquimod propionate cream (Sichuan Med-Shine Pharmaceutical CO., LTD, China), 0.02% clobetasol propionate cream (Shanghai General Pharmaceutical CO., LTD, China), anti-T cell receptor (TCR) γδ antibody conjugated to biotin (Abcam, Cambridge, UK), anti-RORγ antibody (Abcam, Cambridge, UK), anti-CCR6 antibody (Abcam, Cambridge, UK), streptavidin-peroxidase polymer (Sigma, St. Louis, MO, USA), goat anti-rabbit IgG conjugated to biotin (Santa Cruz Biotechnology, USA), REAL™ EnVision™ detection system, peroxidase/DAB+,rabbit/mouse (Dako, Denmark). TRIzol reagent (Invitrogen, Carlsbad, USA), primescript RT reagent kit and SYBR^®^ premix Ex Taq™ II(Takara, Dalian, China). Water was distilled and purified using a Milli-Q Water Purification System (Millipore, USA). Other chemicals were of analytical grade.

### Preparation of Curcumin Gel

The 1% curcumin gel formulation used in this study was based on and improved from a formulation used in a previous study [16]. The composition of the gel is as follows: curcumin 1 g, HPC 3 g, azone 1 g, ethanol (96%) 17 g, and distilled water q.s. to 100 g. HPC powder was added to the distilled water while stirring. The solution was incubated at ambient temperature overnight. The ethanol solution containing curcumin was added to the aqueous solution of HPC, and the resulting mixture was stirred continuously until the gel formed. Azone was incorporated into the gel formulation by solubilization in the ethanol solution of curcumin. This gel formulation of curcumin showed excellent transdermal effects in an in vitro permeation experiment (manuscript in press).

### Mice and Treatment

6–8 weeks old BALB/c mice were purchased from Shanghai SLAC Laboratory Animal Co. Ltd and randomly divided into 5 groups (N = 6). Mice were housed under specific pathogen-free conditions and provided with food and water ad libitum. Based on a previous report [15], the psoriasis-like skin inflammation mouse model was generated by daily topical application of a dose of 20 mg/cm^2^ IMQ cream (5%) on the inside of the right ear for 10 consecutive days. For therapy, the psoriasis-like skin inflammation mouse model was treated twice daily with topical 50 mg/cm^2^ curcumin HPC gel or 40 mg/cm^2^ clobetasol propionate cream. Mice treated similarly with HPC gel (without curcumin and azone) were used as a control group.

### Measurement of Ear Thickness

The ear thickness was measured using a thickness gauge (Guanglu Digital Caliper Manufacturer Co., Ltd, China) at 0, 2, 4, 6, 8, 10 days. The increase in ear thickness was used to indicate the extent of inflammation. Data from three repeat experiments were averaged and used to evaluate epidermal proliferation and inflammation.

### Scoring Severity of Skin Inflammation

To score the severity of the inflammation of the ear skin, an objective scoring system was developed based on the clinical Psoriasis Area and Severity Index (PASI). Erythema, scaling, and thickening were scored independently from 0 to 4 as follows: 0, none; 1, slight; 2, moderate; 3, marked; 4, very marked. The level of erythema was scored using a scoring table with red taints. The cumulative score (erythema plus scaling plus thickening) served to indicate the severity of inflammation (scale 0–12).

### Histology and Immunohistochemistry

At experimental day 10, ear samples from each mouse from each group were fixed in 4% paraformaldehyde and embedded in paraffin. Sections (thickness = 4 µm) were stained using hemotoxylin and eosin (H&E). Antigen retrieval was performed by micro-waving at 100°C at moderate power using a Galanz microwave oven (Galanz model number P70D20P-TF), pausing for 10 min and then micro-waving at 100°C at minimum power. Sections were washed with PBS and incubated with H_2_O_2_ for 10 min to block endogenous peroxidases and then soaked in 5% BSA for 20 min. Sections were then incubated overnight (18 h) at 4°C with the following: anti-mouse CCR6 (diluted 1∶100), anti-mouse TCR γδ (diluted 1∶50) or anti-mouse RORγ (diluted 1∶50). The sections were then incubated with diluted biotin-conjugated goat anti-rabbit IgG (50 min) at 4°C (except sections examining TCR γδ). Finally, the sections were developed using DAB, stained lightly with haematoxylin and eosin and fixed using neutral balata. Isotype controls were used as negative controls, for which Armenian hamster monoclonal IgG (Abcam, Cambridge, UK) and rabbit polyclonal IgG (Abcam, Cambridge, UK) were used. Additional sections were processed without primary antibodies.

TCR γδ-positive skin-tissue cells were counted independently by two persons using an optical microscope (Nikon, Japan) at 400× magnification who were blinded to the parameters of the study [17], [18]. Five fields from each section that did not overlap were randomly selected, and TCR-positive cells were counted and recorded. The average number of TCR γδ-positive cells per high-power field was calculated.

### Western-blot

To determine the level of CCR6 and RORγ expression in the mouse ear samples, the tissues were cleaned using Tris-buffered saline (TBS), cut into pieces and homogenized. Total proteins were extracted as described elsewhere [19]. A total of 20–40 µg of protein was subjected to SDS-PAGE, separated by electrophoresis and transferred to a polyvinylidene difluoride (PVDF) membrane. The membrane was incubated with primary antibody followed by incubation with a species-appropriate secondary antibody coupled to horseradish peroxidase. Bands were visualized using an ECL detection kit.

### Real-time Quantitative PCR

Total mRNA was extracted from whole biopsies of the ear using TRIzol reagent after euthanizing the mice; the mRNA was then transcribed to cDNA. Quantitative real-time PCR was performed using a Light Cyclear-Faster-Start DNA Master SYBER Green I kit (Takara, Dalian, China). The cycling conditions comprised 40 cycles at 95°C for 15 s and 60°C for 30 s using a single fluorescence measurement. Melting curve analysis, for which the temperature was increased from 60°C to 95°C at a heating rate of 0.1°C/s using a continuous fluorescence measurement, revealed a single, narrow peak of suspected fusion temperature. The sequences of the real-time PCR primers were showed in Table 1. Cytokine and GAPDH mRNA levels were calculated relative to amounts of a standard sample, and cytokine mRNA levels were corrected for GAPDH mRNA levels to normalize RNA input.

**Table 1:** Primer sequences of mouse genes examined by quantitative real-time PCR.

Target gene	Primer sequence
IL-17A (S)	5′- CTGCTGAGCCTGGCGGCTAC-3′
IL-17A (AS)	5′- CATTGCGGTGGAGAGTCCAGGG-3′
IL-17F (S)	5′- ACCCGTGAAACAGCCATGGTCAAG-3′
IL-17F (AS)	5′- CCCATGGGGAACTGGAGCGG-3′
IL-22 (S)	5′- CAGCTCCTGTCACATCAGCGGT-3′
IL-22 (AS)	5′- AGGTCCAGTTCCCCAATCGCCT-3′
IL-23 (S)	5′- TCCTCCAGCCAGAGGATCACCC-3′
IL-23 (AS)	5′- AGAGTTGCTGCTCCGTGGGC-3′
TNF-α (S)	5′-GCCCACGTCGTAGCAAACCAC-3′
TNF-α (AS)	5′-GCAGGGGCTCTTGACGGCAG-3′
STAT3 (S)	5′- GAAGCCGACCCAGGTGC-3′
STAT3 (AS)	5′- GTCACGTCTCTGCAGCTTCT-3′
IL-1β (S)	5′- CCCTGCAGCTGGAGAGTGTGGA-3′
IL-1β (AS)	5′- TGTGCTCTGCTTGTGAGGTGCTG-3′
IL-6 (S)	5′- CCTCTCTGCAAGAGACTTCCAT-3′
IL-6 (AS)	5′- AGTCTCCTCTCCGGACTTGT-3′
GAPDH (S)	5′-GGGCTCTCTGCTCCTCCCTGT-3′
GAPDH (AS)	5′-CGGCCAAATCCGTTCACACCG-3′

AS, antisense; S, sense;

### Statistics

All experiments were performed in triplicate using a minimum of three replicates. The means and standard deviations were calculated. The significance of the differences between the treatments was determined using either ANOVA analysis or Wilcoxon scores (rank sums)/Kruskal-Wallis test. A P-value of 0.05 was considered statistically significant.

## Results

### Curcumin Treatment Reduces IMQ-induced Incrassation and Skin Inflammation in Mouse Ear

**Figure 1:**
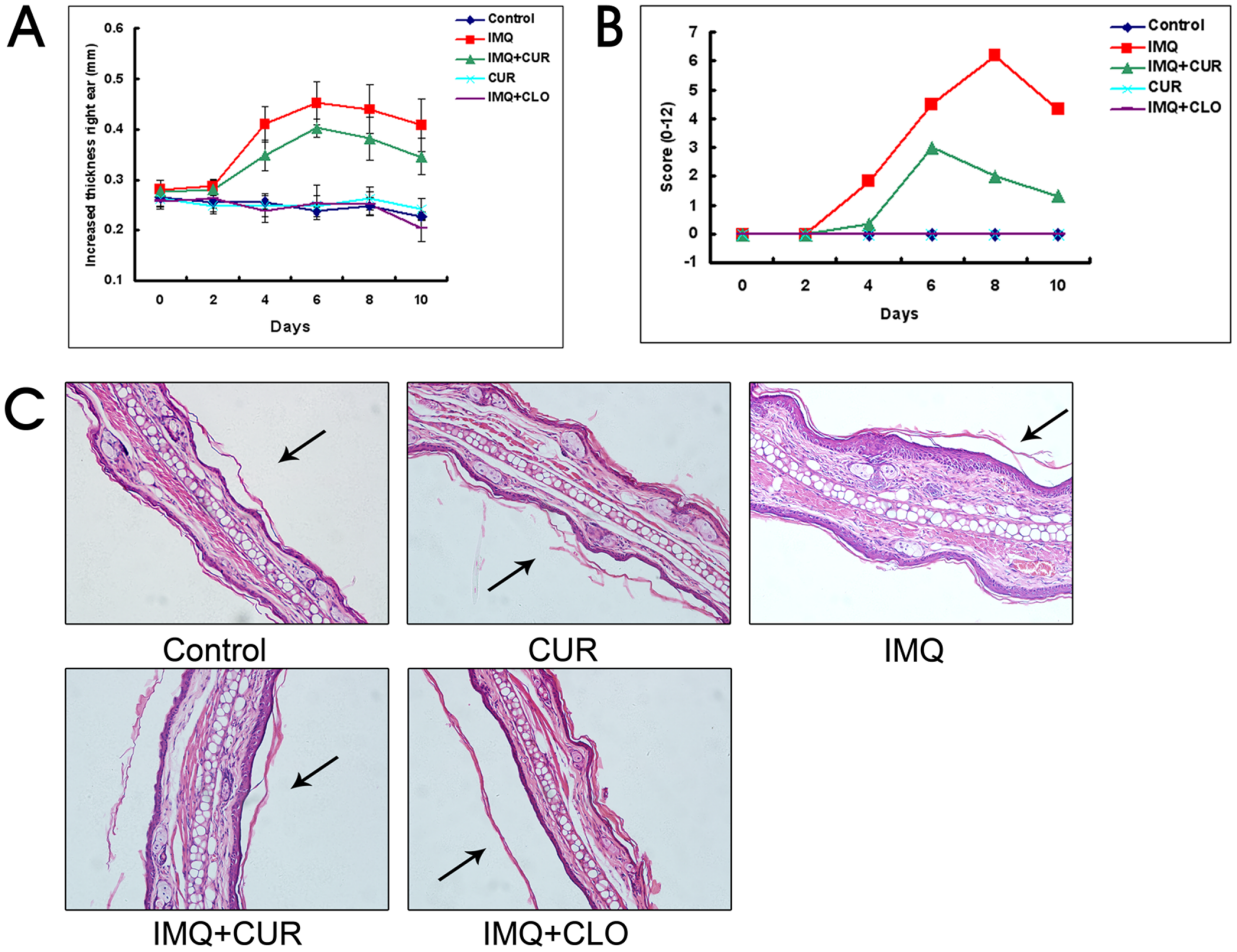
Curcumin inhibited IMQ-induced psoriasis-like inflammation (N = 6). A, The thickness of the right ear skin was measured on the days indicated (Mean±SD). B, Erythema, scaling and thickness of the right ear skin was scored on the indicated days with a scale from 0 to 4. The cumulative score is presented (Mean±SD). C, H&E staining of the mouse ear skin of different treatment groups (200×). Arrowheads denote the inside of the ear skin. CUR, curcumin; CLO, clobetasol.

A summary of the cutaneous inflammation in 5 differently treated groups is shown in Fig. 1. The maximum increase in ear thickness in the IMQ-treated group was 0.452±0.044 mm on day 6, which decreased thereafter to 0.408±0.053 mm on day 10. In contrast, the maximum increase in ear thickness in the curcumin-treated group was 0.403±0.019 mm (*P*<0.05, compared with the IMQ-treated group) on day 6, which decreased to 0.345±0.036 mm on day 10. The ear thickness of the other 3 groups showed no significant changes during the experiment (Fig. 1A).

On day 2–4 after the initiation of IMQ treatment, the ear skin of the IMQ-treated mice began to display signs of thickening, erythema, and scaling (very mild). From days 2–4 onward, inflammation was visible and continuously increased in severity up to days 6–8 and thereafter decreased. Similar results were observed in the curcumin-treated group, but the symptoms were milder than those observed in the IMQ-treated group. No inflammation was visible in the other groups during the experiment. The total scores for all groups in the experiment are depicted in Fig. 1B.

Results from H&E-stained IMQ-treated ear skin showed increased epidermal thickness and subcutaneous tissue compared with the control group. This increased thickness was due to the hyperplasia of basal and suprabasal keratinocytes. Additionally, abnormal keratinocyte differentiation with marked parakeratosis (nuclei in the stratum corneum) was observed. Curcumin inhibited the IMQ-induced increased thickness of both the epidermal and subcutaneous tissue, whereas clobetasol completely inhibited the increase in thickness. No abnormal phenotype was observed in the other two groups, as shown in Fig. 1C.

### Curcumin Decreased the High mRNA Levels of IL-17A, IL-17F, IL-22 and Other Pro-inflammatory Cytokines in Ear Samples of the IMQ-induced Psoriasis-like Mouse Model

**Figure 2:**
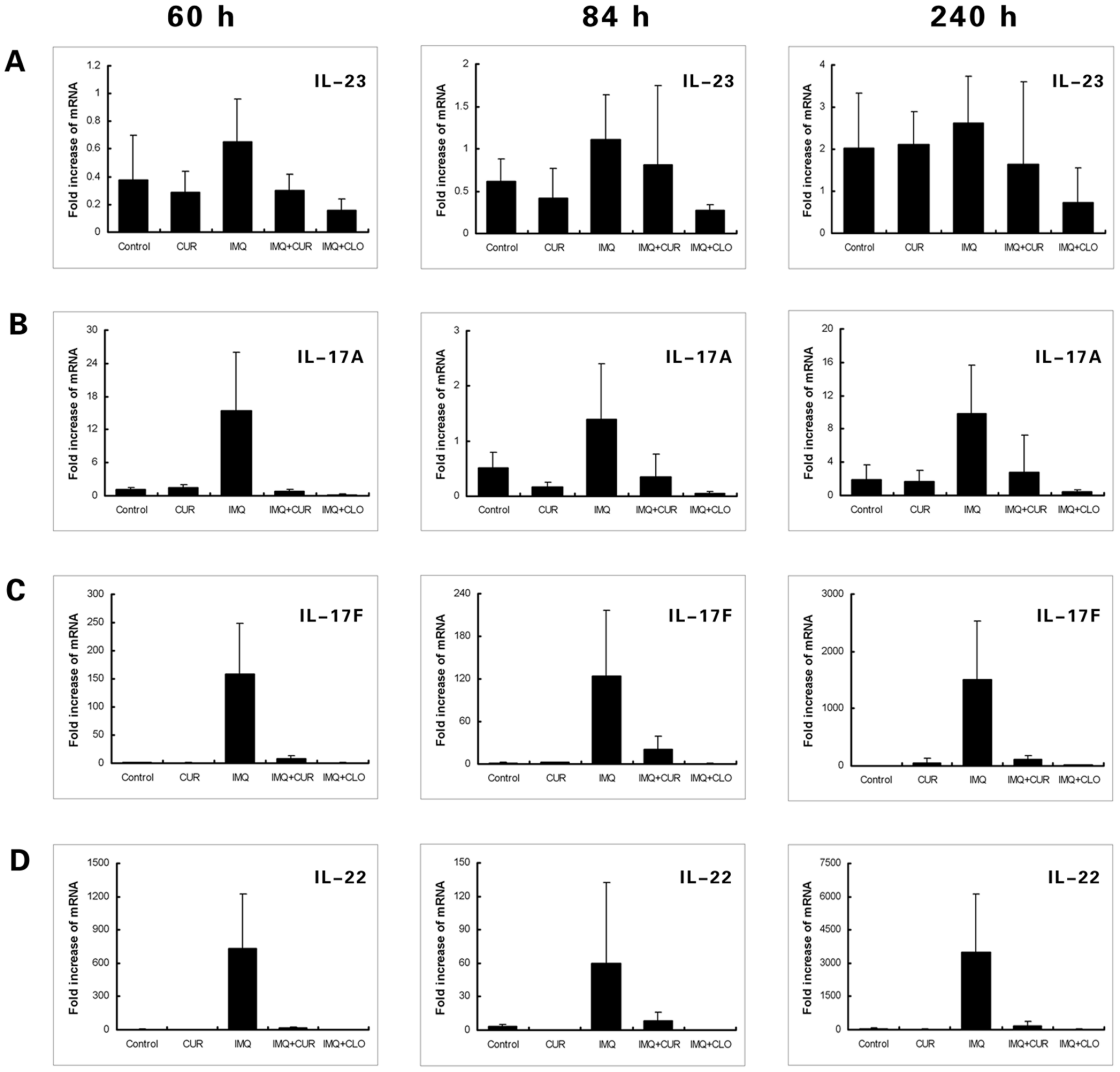
The levels of mRNA measured using real-time PCR (Part 1). The figures show the mean value ± SD (fold) of the measured mRNA in mouse ear tissue of different treatment groups at 60 h, 84 h, and 240 h. A, IL-23. The changes in IL-23 mRNA between groups were statistically insignificant (N = 6). B, IL-17A. The level of IL-17A mRNA in the IMQ-treated group was increased and was inhibited in the CUR-/CLO-treated groups. C, IL-17F. The level of IL-17F mRNA in the IMQ-treated group increased remarkably and was inhibited in the CUR-/CLO-treated groups. D, IL-22. The level of IL-22 mRNA in the IMQ-treated group was increased significantly and was inhibited in the CUR-/CLO -treated groups. CUR, curcumin; CLO, clobetasol.

**Figure 3:**
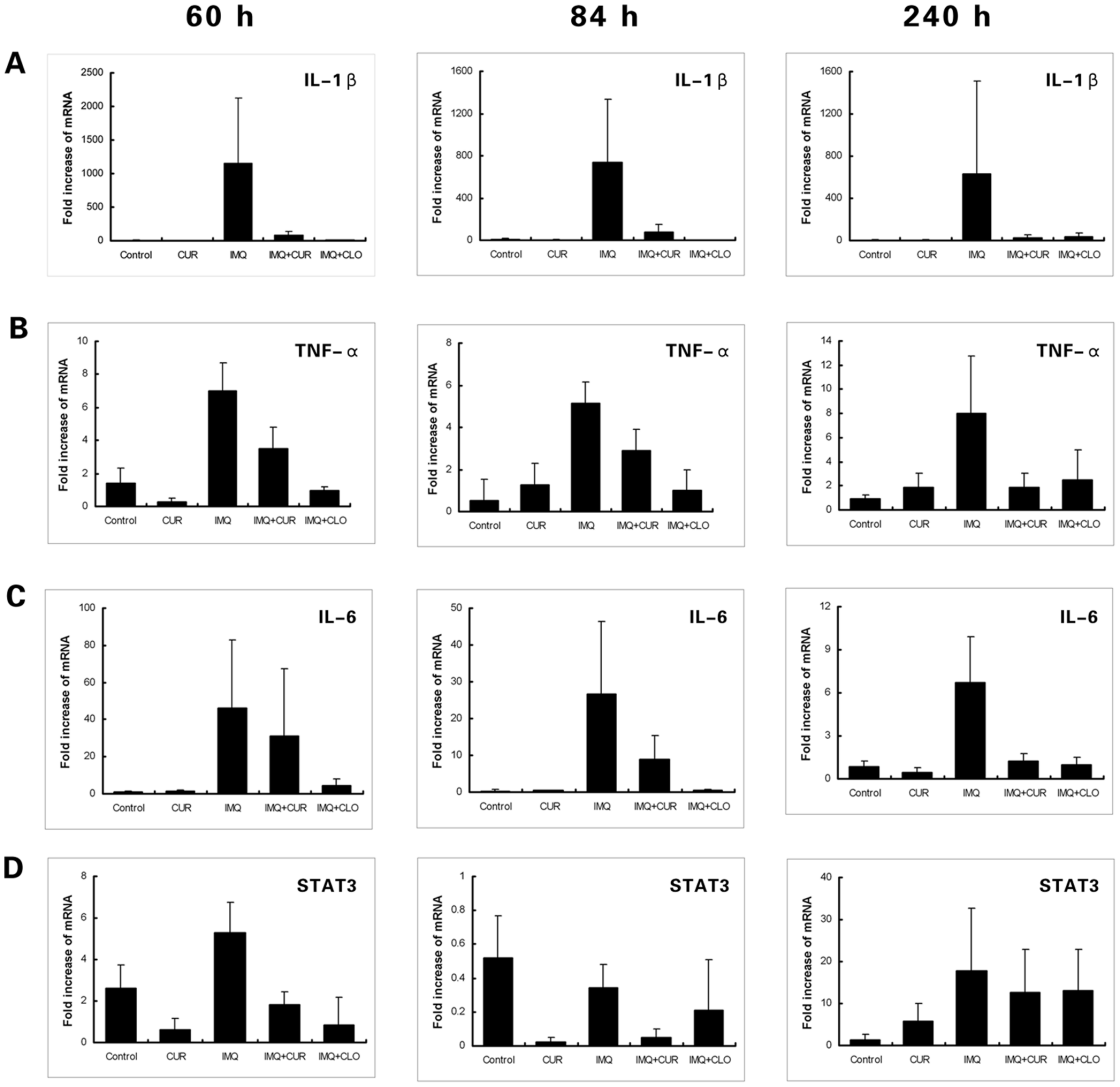
The levels of mRNA measured using real-time PCR (Part 2). The figures show the mean value ± SD (fold) of the measured mRNA in mouse ear tissue of different treatment groups at 60 h, 84 h, and 240 h (N = 6). A, IL-1β. The level of IL-1β mRNA in the IMQ-treated group was sharply increased and was inhibited in the CUR-/CLO-treated groups. B, TNF-α. The level of TNF-α mRNA in the IMQ-treated group was increased and was inhibited in the CUR-/CLO-treated groups. C, IL-6. The level of IL-6 mRNA in the IMQ-treated group was increased and was inhibited in the CLO-treated group. D, STAT3. The level of STAT3 mRNA in all groups except control was increased at 240 h. CUR, curcumin; CLO, clobetasol.

Results from real-time PCR measurements of cytokines in ear tissue are shown in Figs. 2 and 3. In this study, three time points (60, 84, 240 h) were used to test for cytokine mRNA levels. Compared with the control group, the mRNA levels of IL-17A, IL-17F, IL-22, IL-1β, TNF-a and IL-6 were significantly increased (*P*<0.05) in samples from IMQ-treated mouse ear tissue. In contrast, the mRNA levels of IL-17A, IL-17F, IL-22, IL-1β and TNF-a were significantly decreased in the curcumin-treated group when compared with the IMQ-only treated group (*P*<0.05); similar results were observed in the clobetasol-treated group with the addition of IL-6. Moreover, mRNA levels of IL-17F increased more dramatically than IL-17A, indicating that IL-17F may play a more important role than IL-17A in IMQ-induced psoriasis-like inflammation.

Our mRNA results showed that IL-1β was the most significantly increased cytokine compared with TNF-a and IL-6 in the IMQ-treated group; mRNA levels of IL-23 failed to show any significant change in any group. Additionally, our results showed that STAT3 mRNA increased approximately 5-20-fold at 240 h in all experimental groups except the control group.

### The Impact of Curcumin on TCR γδ Positive Cells Proliferation and RORγ/CCR6 Expression in Skin

**Figure 4:**
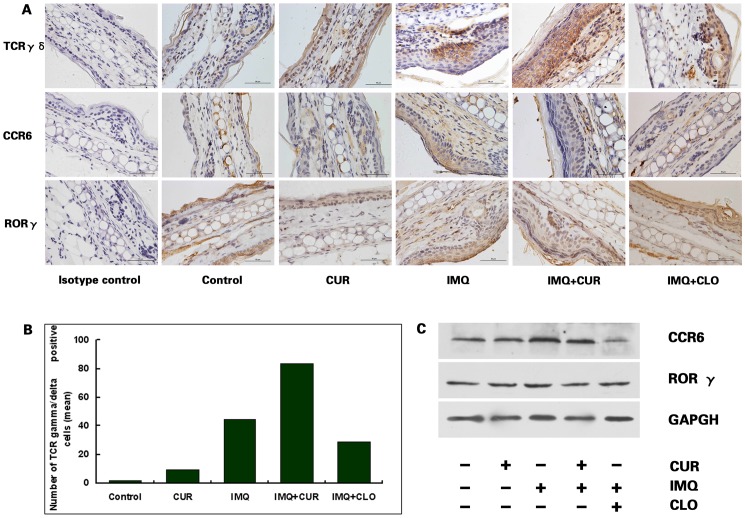
Immunohistochemical staining for TCR γδ, CCR6 and RORγ in skin. A, TCR γδ, CCR6 and RORγ immunohistochemical staining was examined in samples of differently treated mouse ear skin (N = 6). B, The mean number of TCR γδ-positive cells per high power field (N = 6). The differences between groups and between any two groups were significant (Kruskal-Wallis test/Wilcoxon Scores test, *P*<0.05). C, CCR6 and RORγ expression was detected using western blotting. CCR6 expression was increased in IMQ-treated mice and was inhibited by both curcumin and clobetasol. The results shown are representative of 3 independent experiments. CUR, curcumin; CLO, clobetasol.

Immunohistochemical results from IMQ-treated ear skin showed increased TCR γδ-positive cells in skin (shown in Fig. 4 A). Interestingly, TCR γδ-positive cells were remarkably increased after curcumin treatment in both IMQ-treated and control mouse epidermis (shown in Fig. 4 B). These results may provide evidence to suggest that curcumin can promote epidermal TCR γδ-positive cell proliferation independent of IL-23. Although TCR γδ-positive cells were decreased after clobetasol treatment, there were many more than in the control group (*P*<0.05).

Results from western blotting of IMQ-treated ear skin showed increased CCR6 expression when compared with other groups; both curcumin and clobetasol inhibited the IMQ-induced up-regulation of CCR6 expression (shown in Fig. 4 C). In all groups, RORγ expression failed to show any significant difference (shown in Fig. 4 C).

## Discussion

A functional role for the IL-23/IL-17A axis in the pathogenesis of psoriasis was suggested recently [4]. IMQ is a ligand for Toll-like receptor (TLR) and has been used for the topical treatment of genital and perianal warts caused by human papilloma virus, [20] although it can exacerbate psoriasis in well-controlled patients [21], [22]. Investigators confirmed that IMQ-induced psoriasis-like inflammation is mediated by the IL-23/IL-17A axis [15]. Topical application of IMQ on mouse skin leads to the rapid proliferation of plasmacytoid dendritic cells [23] and stimulates keratinocytes to increase cytokine production [24], [25]. These effects in the mouse skin closely resemble human plaque-type psoriasis with respect to erythema, skin thickening, scaling, epidermal alteration (acanthosis, parakeratosis), and so on. Notably, although some of the effects described above were observed in our study, such as skin thickening and inflammation, those symptoms tended to decline after 6 days of IMQ treatment. We believe that the symptoms of the IMQ-induced psoriasis-like model are unstable due to the adaptive reaction of the skin to IMQ stimulation. It is well know that there is a paucity of animal psoriasis models available particularly the one which can imitate pathogenesis of psoriasis. The pathogenesis of IMQ-induced psoriasis-like inflammation was similar with psoriasis. But we suggest it would be prudent to use the IMQ-induced psoriasis-like model in the evaluation of therapies for chronic psoriasis.

In this study, we observed that curcumin inhibited the increase in skin thickness and inflammation in IMQ-treated mouse ear skin. The increase in thickness is due to hyperplastic basal, suprabasal keratinocytes and subcutaneous tissue in the skin. Although the abnormal keratinocyte differentiation with marked parakeratosis was characteristic of this model, it remains unclear whether curcumin can impact this characteristic.

IL-23 has had a known association with psoriasis since its discovery [26], [27]. In our study, IL-23 did not show any significant changes in any group, including the IMQ-treated group. But curcumin decreased the mRNA levels of IL-1β, TNF-a and IL-6 significantly. IL-1β and IL-6 cooperate with IL-23 in IL-17A production in dermal γδ T cells [2], and are critical auxiliary elements in the IL-23/IL-17A/IL-22 axis. Recent studies have shown that IL-1 signaling represents a key step in IL-17A-mediated autoimmune diseases, including psoriasis [28]. Upon IL-1β binding to IL-1R1, the downstream signaling cascade is initiated, followed by the activation of the NF-κB, JNK and p38 MAPK pathways. Daisuke Tsuruta [29] has reviewed in detail the NF-κB link to keratinocytes and lymphocytes in the pathogenesis of psoriasis. Curcumin can inhibit the activation of NF-κB by inhibiting IκB phosphorylation and degradation. Thus, when production and signaling of IL-1β/IL-6 are suppressed by curcumin, IL-17A production must correspondingly decline following IL-23 induction.

The IL-17 family of cytokines comprises six family members of varying homology and function: IL-17A (also called IL-17), IL-17B, IL-17C, IL-17D, IL-17E, and IL-17F [30]–[32]. When the IL-17A receptor is engaged by its ligand, it induces the activation of the NF-κB and JUN amino-terminal kinase (JNK) signaling pathways in a TNFR-associated factor 6 (TRAF6)-dependent manner [33]. IL-17A also synergizes with other cytokines, such as IL-1β, IL-6 and TNF-a, that promote the activation of tissue-infiltrating neutrophils. Our results showed that curcumin sharply reduced the increase in IL-17A and IL-17F expression induced by IMQ in mouse skin. The orphan nuclear receptor RORγ, which is activated by IL-6 and TGF-β, is required for the expression of IL-17A both *in vitro* and *in vivo* [34]. Because we have no evidence of curcumin’s influence on RORγ expression, we infer that curcumin down-regulated IL-17A/IL-22 expression indirectly by inhibiting IL-1β/IL-6 production. And interestingly, IL-17F expression increased more markedly than IL-17A in IMQ-treated mouse skin, indicating that IL-17F may have a greater contribution to IMQ-induced psoriasis-like inflammation. The expression of IL-22 in IMQ-induced psoriasis-like inflammation was also markedly down-regulated by curcumin. It is notable that IL-17A often functions cooperatively with IL-22.

Increased activation of STAT3 exists in keratinocytes from human psoriatic lesions [35]. STAT3 has a pathogenetic role by altering the proliferative and differentiation processes in keratinocytes, and inducing inflammatory molecules [36]. The increased levels of cytokines and growth factors contribute to promote STAT3 activation in psoriatic lesions. For instance, IL-22 potently triggers STAT3 [36]. In this study the expression of STAT3 increased after 240 h in all groups except the control. Thus far, no evidence supports an inhibitory or enhancement effect of curcumin on the expression of STAT3. We inferred that the adjuvant material or transdermal enhancers (both curcumin gel and clobetasol propionate cream contain azone) may have contributed to the up-regulation in STAT3 expression. Whether this effect poses a potential risk for psoriasis therapy remains unknown.

**Figure 5:**
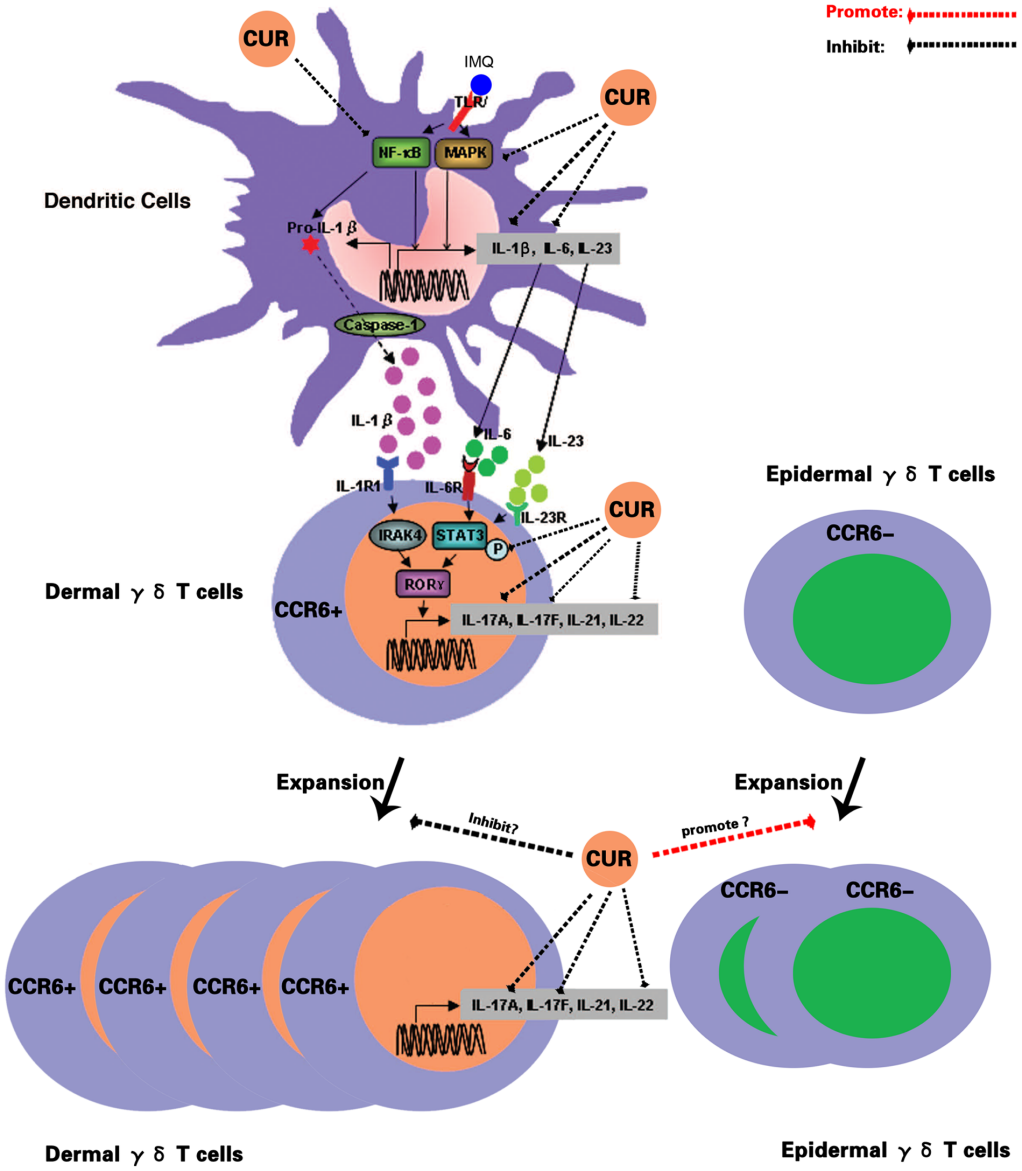
The possible targets impacted by curcumin in the IL-23/IL-17A axis. **CUR, curcumin.**

Recent studies have indeed shown that γδ T cells are capable of producing both IL-17A and IL-17F [37]. In murine skin, γδ T cells are abundant and act as dendritic epidermal T cells for local immune surveillance. And IL-23 is essential for both maintaining dermal γδ T cell homeostasis and for regulating their differentiation program but not for epidermal γδ T cells [2]. We found epidermal γδ T cells were remarkably increased following curcumin/IMQ treatment in mouse epidermis. Furthermore, although epidermal γδ T cells were reduced after clobetasol treatment, the levels remained higher than in the control group. All of the above serve to indicate that curcumin may promote epidermal γδ T cell proliferation. It has confirmed that CCR6 is the optimal cell surface marker for IL-17A-producing cells [38]. Moreover, dermal γδ T cells but not epidermal γδ T cells expressed CCR6 constitutively [2]. Our results showed that CCR6 expression increased in IMQ-treated ear skin and that curcumin/clobetasol decreased its expression. This result indicated that although curcumin promoted epidermal γδ T cell expansion, it did not do so in dermal γδ T cells. So IL-17A and IL-17F production both failed to increase after curcumin treatment and, if anything, they decreased. The possible multiple targets of curcumin in the IL-23/IL-17A axis are summarized and shown in Fig. 5.

Steroid hormone products, including clobetasol, have been approved for psoriasis therapy by the FDA. Although clobetasol, as a positive control in this study, showed a more powerful effect, curcumin also showed inhibition of IMQ-induced psoriasis-like inflammation. It is well known that topical corticosteroids are associated with serious adverse reactions. Fortunately, curcumin does not.

Despite its demonstrated efficacy, the limited bioavailability of curcumin continues to be an insurmountable barrier and obstacle; its low bioavailability makes it extremely difficult for curcumin to play its expected role in target cells. With regard to skin, the most superficial organ, it is possible for curcumin to reach target cells at high concentrations via topical application. Thus far, the research on curcumin topical application transdermal was scarce and was puzzled by three problems: low transdermal delivery efficiency, poor stability and staining clothes/skin orange which limits its use in the consumer markets. Fortunately, we solved one by Azone to increase curcumin transdermal delivery markedly. It is on that basis, we discovered that curcumin can suppress inflammation in IMQ-induced psoriasis-like mice model and decrease the cytokines production, such as IL-17A, IL-17F, IL-22, IL-1β and TNF-α. Nevertheless, in this first step, we hope that these new data evoke an encouraging perspective for the therapeutic application of curcumin.
